# Epidemiological analysis and potential factors affecting the 2022–23 Crimean-Congo hemorrhagic fever outbreak in Iraq

**DOI:** 10.1093/eurpub/ckae147

**Published:** 2025-01-13

**Authors:** Chiori Kodama, Riyadh Abdulameer Alhilfi, Ihab Aakef, Adnan Khamasi, Sinan Mahdi, Hameeda Mohammed Hasan, Raghad Ibrahim Khaleel, Mazin Mahdi Naji, Noor Khalid Esmaeel, Sundus Haji-Jama, Anais Legand, Olivia Keiser, Isabella Eckerle, Pierre B H Formenty

**Affiliations:** World Health Organization, Eastern Mediterranean Regional Office, Cairo, Egypt; Global Health Institute, University of Geneva, Geneva, Switzerland; Directorate of Public Health, Ministry of Health, Baghdad, Iraq; Communicable Disease Control Center, Ministry of Health, Baghdad, Iraq; World Health Organization, Country Office for Iraq, Baghdad, Iraq; Communicable Disease Control Center, Ministry of Health, Baghdad, Iraq; Communicable Disease Control Center, Ministry of Health, Baghdad, Iraq; Central Public Health Laboratory, Ministry of Health, Baghdad, Iraq; Central Veterinary Laboratory, Ministry of Agriculture, Baghdad, Iraq; Central Veterinary Laboratory, Ministry of Agriculture, Baghdad, Iraq; World Health Organization, Eastern Mediterranean Regional Office, Cairo, Egypt; World Health Organization, Headquarters, Geneva, Switzerland; Global Health Institute, University of Geneva, Geneva, Switzerland; Geneva Centre for Emerging Viral Diseases, University Hospitals of Geneva and Faculty of Medicine, University of Geneva, Geneva, Switzerland; World Health Organization, Headquarters, Geneva, Switzerland

## Abstract

Crimean-Congo hemorrhagic fever (CCHF) is an acute tick-borne disease with a case fatality rate of up to 40% in humans, posing a significant health threat. This study investigates the 2022–23 CCHF outbreaks in Iraq, the highest recorded to date, and analyzes potential factors at the human–animal–environmental interface. Data from the Iraqi government, the World Health Organization, and the World Bank were used to analyze CCHF trends and affecting factors. This included epidemiological reports, clinical data, tick infestation and seroprevalence studies, and climate data. Descriptive and statistical analyses examined case trends, geographic and demographic characteristics, clinical manifestations, risk factors, seasonal patterns, and influencing factors. A sudden rise in CCHF cases began in southern Iraq in April 2022 and expanded across all governorates, with a shift toward urban areas. Higher incidence was observed among males, aged 25–44, and those involved in slaughtering. The most common clinical manifestation was fever (97%), followed by hemorrhagic symptoms (54%). Bleeding from the gums or mouth and subcutaneous bleeding were more frequent in patients with fatal outcomes. Seasonal patterns showed peaks during spring and fall, correlating with tick activity and potentially exacerbated by climate change. Tick infestation and seroprevalence studies indicated a high prevalence of Hyalomma ticks and CCHF seropositivity among domestic animals in southern Iraq (60%), consistent with the distribution of CCHF human cases. Iraq’s ongoing CCHF outbreak demands multidisciplinary One Health strategies. The Iraqi government has adopted such a control strategy, contributing to regional and global efforts to enhance pandemic preparedness.

## Introduction

Crimean-Congo hemorrhagic fever (CCHF) is caused by a tick-borne virus, Orthonairovirus hemorrhagiae (Crimean-Congo hemorrhagic fever virus [CCHFV]), of the Orthonairovirus genus of the Nairoviridae family. The CCHFV causes severe viral hemorrhagic fever outbreaks, with a case fatality rate (CFR) of 10%–40% [1–3]. Globally, an estimated 3 billion people are at risk of CCHFV infection, and 10 000–15 000 CCHFV infections occur each year in many countries across Africa, Asia, the Balkans, and the Middle East [1–3] (Supplementary Fig. S1). However, the true burden of CCHF remains unknown due to multiple factors, including insufficient One Health research and operations jointly by human and veterinary sectors, fragmented surveillance and reporting systems, limited laboratory capacity for confirmation, insufficient capacity among healthcare workers to suspect and provide timely treatment, and lack of awareness and preventative measures among communities. CCHF poses a global health risk due to its epidemic potential, high fatality rate, and lack of specific treatments or vaccines [4–10].

Ixodid (hard) ticks, especially those of the genus Hyalomma, are a reservoir and a vector for the CCHF virus with a transovarian cycle. Wild and domestic animals, such as cattle, goats, sheep, and hares, serve as amplifying hosts for the virus [4, 5]. The infected animals are asymptomatic, while the disease in humans can be severe, with a CFR of up to 50% [6–8]. Transmission to humans occurs through exposure to infected tick bites, contact with infected animal tissues, blood, or fresh meat, and from infected humans to others by contact with infectious blood or body fluids [6–9]. Nosocomial CCHF infection can occur in healthcare settings lacking adequate infection prevention and control measures [6–10].

### Regional distribution of CCHF

In the World Health Organization Eastern Mediterranean Region, sporadic human cases and outbreaks of CCHF have been reported from Afghanistan, Iran, Iraq, Kuwait, Oman, Pakistan, Saudi Arabia, Sudan, and the UAE [2, 9, 11–15]. Moreover, serological studies among livestock have identified the presence of the disease in Egypt, Somalia, and Tunisia [2, 9]. The disease is reportedly endemic in Afghanistan, Iran, Iraq, Oman, and Pakistan, particularly in the areas bordering these countries where frequent movement of nomads with their animals is concentrated. Trade in animals within those countries, and between Afghanistan, Iran, Iraq, and Pakistan, is thought to play a major role in the spread of CCHFV among people who handle animals, slaughter infected animals, and/or come into close contact with ticks or CCHF patients.

Iraq reported its first CCHF case in 1979. Since then, Iraq has annually reported between 5 and 40 cases, peaking at 48 in 1996 [2, 9, 16] (Supplementary Fig. S2). In 2022 and 2023 [17], Iraq reported two large outbreaks of CCHF, detailed in this study (Table 1). As a systematic and comparative analysis of the CCHF human and animal data for 2022–23, as well as the driving forces behind the sudden increase in cases starting in 2022, had never been conducted, this study collected data from both human and veterinary sectors and conducted a descriptive analysis of the collected data. At the later stage of research, the study also analyzed potential factors affecting the 2022–23 outbreaks of CCHF cases in Iraq.

**Table 1.** Number of CCHF cases and CFR reported by Iraqi governorates and comparison between 2022 and 2023^a^

Province	2022–23		Increase/decrease^b^ in 2023 compared with 2022		2023		2022	
	Cases	CFR^c^ (%)	No. of cases	CFR^c^ (%)	No. of cases	CFR^c^ (%)	No. of cases	CFR^c^ (%)
Al-Anbar	3	0	100		2.0	0	1	0
Al-Sulaymaniyah	4	25			4.0	25	0	
Babil	47	11	−4	57	23.0	13	24	8
Baghdad	117	17	233	−10	90.0	17	27	19
Basra	105	14	353	44	86.0	15	19	11
Diwanya	41	15	41	42	24.0	17	17	12
Diyala	23	30	260	−63	18.0	22	5	60
Duhok	8	0	67		5.0	0	3	0
Erbil	17	41	367	29	14.0	43	3	33
Kerbala	28	21	−35	55	11.0	27	17	18
Kirkuk	18	17	400	−60	15.0	13	3	33
Maysan	76	8	5	−5	39.0	8	37	8
Muthanna	55	18	75	−62	35.0	11	20	30
Najaf	22	18	240	−71	17.0	12	5	40
Ninewa	22	18	44		13.0	31	9	0
SalahAl-Din	12	25	400	−90	10.0	10	2	100
ThiQar	304	18	−12	−53	142.0	11	162	24
Wasit	65	8	50	−56	39.0	5	26	12
Total	967	16	54	−27	587.0	14	380	19

a Data source: Ministry of Health Iraq/Communicable Disease Control Center.

b Increase/decrease in 2023 compared with 2022 = (CFR2023 − CFR2022)/CFR 2022.

c CFR = case fatality rate. Decimal points are rounded up in the calculations.

## Methods

### Study design

This study is designed as a descriptive and statistical analysis, focusing on the geographical and seasonal epidemiology, profile of the affected population, clinical data between survived and deceased, tick infestation, seroprevalence in domestic animals, and climate data as potential factors affecting CCHF outbreaks with a focus on January 2022 to December 2023.

### Data source and collection

Data were sourced from the Ministry of Health Iraq, the Ministry of Agriculture Iraq, the World Health Organization, and the Climate Change Knowledge Portal by the World Bank [18].

#### Human case reports

The study population included all individuals confirmed with CCHF reported to the Ministry of Health Iraq/Communicable Disease Control Center/Central Public Health Laboratory from 1 January 2022 to 31 December 2023. Laboratory confirmation for CCHFV was done by Reverse transcription polymerase chain reaction (RT-PCR) and/or IgM according to the national guidelines. Case data, including demographics, exposure history, clinical outcomes, laboratory confirmation, and other necessary information, were collected through national health records and enhanced national surveillance by adding key indicators. Certain indicators were collected via audit from patients or their families in cases where the patient was unable to respond. Suspected cases from 2022 to 2023 were excluded from the study due to observed inconsistencies in the suspected case definition, particularly in 2022.

#### Animal studies

Two studies were conducted by the Ministry of Health/Ministry of Agriculture/Central Veterinary Laboratory in 2023. Domestic animals (cattle, sheep, goats, and buffaloes) were sampled across all governorates to assess tick infestation rates and CCHFV seroprevalence (CCHF IgG). The study aimed to understand the transmission dynamics between animals and humans.

#### Climate data

Average mean surface air temperatures were obtained from the Climate Change Knowledge Portal by the World Bank [18]. The period analyzed spanned from 1 January 1951 to 31 December 2020, focusing on trends in climate change that could influence tick vector habitats and behaviors.

### Data analysis

#### Descriptive and statistical analysis of human and animal data and CCHF trends over time

Epidemiological data on confirmed CCHF human cases, including demographics, occupational exposures, clinical presentations, and outcomes, were analyzed using descriptive statistics. Frequencies, percentages, and rates were calculated to describe the distribution of cases over time and across different regions and populations.

The study also included a descriptive analysis of tick infestation rates and seroprevalence of CCHFV among domestic animals based on field research conducted within the relevant geographic regions. Seroprevalence data from domestic animals were further analyzed to map the geographical distribution of seropositive animals in comparison to the distribution of human cases.

CCHF trends were analyzed descriptively and displayed graphically. The analysis also attempted to identify patterns and shifts in climate conditions that could influence tick populations and, consequently, the transmission of CCHFV to humans.

### Ethical considerations

The data involving human case reports were anonymized and provided under the supervision of the Ministry of Health, Iraq, with ethical approval granted.

## Results

From 1 January to 31 December 2022, Iraq reported 380 laboratory-confirmed CCHF cases, including 74 deaths, with a CFR of 19%. A total of two healthcare workers were reported to be infected in 2022. From 1 January to 31 December 2023, Iraq reported a total of 587 laboratory-confirmed cases, including 83 deaths, with an overall CFR of 14%, and five healthcare workers were reported to be infected with CCHFV. In 2023, the number of reported confirmed cases increased by 54% overall, and 27% decreased in CFR compared to 2022 (95% CI, *P* = .009) (Table 1).

### CCHF geographical distribution in Iraq

Iraq is constituted by 19 governorates, and CCHF outbreaks emerged from the southern governorates. In 2022, a total of five governorates in the south reported 276 (83%) of the overall 380 confirmed cases, with 162 confirmed cases reported from Thi-Qar (43%), 37 from Maysan (10%), 27 from Baghdad (7%), 26 from Wasit (7%), and 24 from Babil (6%). While in 2022, Sulaymaniya in the northern Iraq was the only governorate free of CCHF, in 2023, all 19 governorates, including Sulaymaniya, reported a total of 587 CCHF-confirmed cases.

In 2023, six southern governorates reported 402 (73%) of the overall 587 confirmed cases, with 142 confirmed cases reported from Thi-Qar (24%), 90 from Baghdad (15%), 86 from Basra (15%), 39 from Maysan and Wasit (7% each), and 35 from Al-Muthana (6%). In all but three governorates, an increase in the number of confirmed cases, ranging from 41% to 400%, was reported in 2023 compared to 2022. Detailed numbers of CCHF cases and CFR reported by Iraqi governorates, and a comparison between 2022 and 2023, are shown in Table 1 and Supplementary Fig. S3.

### Rural versus urban settings

For the analysis of rural and urban settings, we used 919 CCHF-confirmed cases with type of settings reported between 2022 and 2023 (Supplementary Fig. S4). In 2022, 242 cases (64%) were reported to live in rural and semi-urban settings and 138 (36%) in urban settings. In 2023, 309 cases out of 539 with available data (57%) were reported to live in rural or semi-urban settings and 217 (40%) in urban settings.

### Distribution of cases by occupation and type of exposure

From the same sample group, occupation was recorded for 915 cases. Occupations were categorized into main categories including “housewife,” assuming duties including preparation of meat, contact with animals, and with wildlife, with 322 cases (35%); “butcher/slaughter,” assuming contact with animals and their products, with 136 cases (15%); “farmer,” assuming contact with wildlife and ticks, with 32 cases (3%); “livestock owner,” assuming contact with animals, with 80 cases (9%); “health workers,” assuming potential contacts with patients, with 7 cases (1%); and “student or children,” with 95 cases (10%). All other occupations (with no obvious risk of exposure) were grouped under “others,” with 243 cases (27%) (Table 2).

**Table 2.** Distribution of CCHF cases in Iraq per reported type of exposure and occupation^a^

Reported exposure(s)	All		Occupation														
			Butcher		Farmer		Health worker		Housewife		Livestock animal		Others		Student/child		*P*-value^b^
*n*, %	915		136	15%	32	3%	7	1%	322	35%	80	9%	243	27%	95	10%	
Count of exposure																	<.001
0	134	15%	5	4%	9	28%	0	0%	28	9%	8	10%	60	25%	24	25%	
1 or 2	519	57%	52	38%	13	41%	7	100%	213	66%	40	50%	145	60%	49	52%	
3 and more	262	29%	79	58%	10	31%	0	0%	81	25%	32	40%	38	16%	22	23%	
Animal in house																	<.001
Yes	494	54%	83	61%	18	56%	1	14%	175	54%	69	86%	100	41%	48	51%	
No	416	45%	53	39%	14	44%	6	86%	144	45%	10	13%	142	58%	47	49%	
Missing	5	1%							3	1%	1	1%	1	0%			
Slaughtering																	<.001
Yes	442	48%	119	88%	12	38%	0	0%	123	38%	31	39%	96	40%	61	64%	
No	467	51%	17	13%	20	63%	7	100%	195	61%	48	60%	146	60%	34	36%	
Missing	6	1%							4	0.01242	1	1%	1	0%			
Tick bite																	.001
Yes	177	19%	20	15%	11	34%	2	29%	68	21%	25	31%	32	13%	19	20%	
No	732	80%	116	85%	21	66%	5	71%	250	78%	54	68%	210	86%	76	80%	
Missing	6	1%							4	1%	1	1%	1	0%			
Raw meat						0%											<.001
Yes	542	59%	124	91%	19	59%	2	29%	206	64%	36	45%	113	47%	42	44%	
No	366	40%	12	9%	13	41%	5	71%	112	35%	43	54%	128	53%	53	56%	
Missing	7	1%							4	1%	1	1%	2	1%			
Contact with cases																	.008
Yes	33	4%	3	2%	1	3%	3	43%	14	4%	1	1%	6	2%	5	5%	
No	869	95%	133	98%	31	97%	4	57%	302	94%	78	98%	232	95%	89	94%	
Missing	13	1%							6	2%	1	1%	5	2%	1	1%	

a Data source: Ministry of Health Iraq/Communicable Disease Control Center. *N* = 915 CCHF-confirmed cases reported from 2022 to 2023, with data available for occupation and exposure type.

b Fisher’s exact test.

Table 2 includes possible exposures recorded and the presence of an animal at home, slaughtering history in the past 2 weeks, contact with raw meat in the past 2 weeks, tick bite, and contact with a suspected or confirmed CCHF patient. A total of 519 patients (57%) reported one or two types of exposure, and 262 (29%) reported three or more types of exposure. The most commonly reported exposure was contact with raw meat (542, 59%), animals in the house (494, 54%), and history of slaughtering (442, 48%). The count of types of exposure varied across occupations, with butchers and livestock owners more likely to have three or more types of exposure. Butchers were more likely to have animals in the house, a history of slaughtering, and contact with raw meat, while livestock owners were more likely to have animals in the house and report tick bite history. Tick bite history and contact with a suspected or confirmed CCHF case were only reported for 177 cases (19%) and 33 cases (4%), respectively.

### Gender and age group distribution

Of all CCHF-confirmed cases reported from 2022 to 2023, a total of 904 with complete line list data were further analyzed. Of these, 371 cases (41%) were female and 533 (59%) were male. The CFR for both genders was 15%. For both males and females, the most affected age group was 25–44 years old, with 156 females and 227 males (42%) (Table 3 and Supplementary Fig. S5). Among males, the second most affected age group was 15–24 years old with 147 cases (27%), while among females, the second most affected age group was 45–64 years old with 105 cases (28%; *P* = .002). The CFR was higher among people over 65 years old, with 11 deaths out of 53 cases (21%; *P* = .005).

**Table 3.** Description of CCHF-positive cases in Iraq by disease outcome^a^

			Disease outcome				
	All		Recovered		Deceased		
	*n*	%	*n*	%	*n*	%	*P*-value^b^
CCHF-confirmed cases	904		762	84	142	16	
Sex							1
Female	371	41	313	41	58	41	
Male	533	59	449	59	84	59	
Age group (years)							.03
1–4	2	0	2	0	0	0	
5–14	31	3	31	4	0	0	
15–24	214	24	188	25	26	18	
25–44	383	42	316	41	67	47	
45–64	221	24	183	24	38	27	
≥65	53	6	42	6	11	8	
Fever							.5
Yes	874	97	738	97	136	96	
No	30	3	24	3	6	4	
Any bleeding signs?							.004
Yes	485	54	393	52	92	65	
No	419	46	369	48	50	35	
Count of bleeding signs							<.001
0	419	46	369	48	50	35	
1–2	355	39	304	40	51	36	
3 and more	130	14	89	12	41	29	
Type of bleeding							
At injection site							<.001
Yes	247	27	186	24	61	43	
No	656	73	575	76	81	57	
Missing	1		1		0		
Bleeding from gums or mouth							<.001
Yes	159	18	118	15	41	29	
No	745	82	644	85	101	71	
Missing	0		0		0		
Epistaxis^c^							.8
Yes	122	14	102	13	20	14	
No	780	86	658	87	122	86	
Missing	2		2		0		
Injected conjunctiva							1
Yes	42	8	37	8	5	7	
No	482	92	419	92	63	93	
Missing	380		306		74		
Bleeding from orifice							.002
Yes	129	14	96	13	33	23	
No	772	86	663	87	109	77	
Missing	3		3		0		
Subcutaneous bleeding							<.001
Yes	224	25	169	22	55	39	
No	680	75	593	78	87	61	
Missing	0		0		0		
Other type of bleeding^d^							.3
Yes	65	16	49	15	16	20	
No	344	84	280	85	64	80	
Missing	495		433		62		

a Data source: Ministry of Health Iraq/Communicable Disease Control Center. *N* = 904 CCHF cases with known outcome, reported from 2022 to 2023.

b Fisher’s exact test.

c Data not recorded in 2022. Missing data excluded from proportion.

d Other type of bleeding included: hematemesis, hematuria, melena, vaginal bleeding, or not specified.

### Clinical presentations

Clinical presentations and disease outcomes were recorded for 904 confirmed cases, including variables such as fever and different types of bleeding (Table 3). Other symptoms were not systematically recorded and could not be analyzed in this study. Fever was most commonly reported in both recovered and deceased patients, with a total of 893 cases (97%). Up to seven different types of bleeding were reported and included: bleeding at the injection site, bleeding from the mouth or gums, epistaxis, injected conjunctiva, bleeding from an orifice, subcutaneous bleeding, and other types of bleeding, including hematemesis, hematuria, melena, vaginal bleeding, or not specified. A total of 485 cases (54%) reported at least one bleeding sign. The most commonly reported bleeding signs were bleeding at the injection site in 247 patients (27%), followed by subcutaneous bleeding in 224 patients (25%). Cases with fatal outcome were more likely to report any type of bleeding signs, to have a higher number of bleeding signs, and to report bleeding at injection sites, bleeding from the gums or mouth, bleeding from an orifice, and subcutaneous bleeding than recovered cases (Supplementary Fig. S6). No significant differences were observed among recovered and deceased for reporting epistaxis and injected conjunctiva.

### The seasonality of CCHF incidence in Iraq

The trend of CCHF cases in Iraq by month from 2022 to 2023 shows a seasonal pattern, between May and November, and peaking from May to July (Supplementary Fig. S7), but cases are observed year-round.

### Tick infestation on domestic animals

The investigation of tick infestation on domestic animals in Iraq was conducted by the Ministry of Health Iraq, Communicable Disease Control Center at the end of 2023. The result showed that various domestic animals were infested with ticks to different extents (Supplementary Fig. S8). Among the analyzed animals, the highest infestation was among sheep, with 86% being infested with ticks, followed by cows (71%), goats (39%), and buffaloes (35%).

### CCHF seroprevalence study on domestic animals

In the quest for CCHF infection among domestic animals, the Ministry of Agriculture Iraq conducted a CCHFV seroprevalence study on domestic animals at the end of 2023. The sample was extracted from domestic animals from 15 governorates and tested for CCHF IgG. A total of 1889 cattle and buffaloes were tested, and 692 were positive (average positivity rate [PR]: 37%). For sheep and goats, a total of 2078 were tested, and 363 were positive (average PR: 17%) (Table 4). Based on this study, a comparative analysis was made between CCHFV serological PR in domestic animals and CCHF-confirmed human cases by governorates in 2023 (Table 4 and Supplementary Fig. S9). The highest serological PR in animals was found in Thi-Qar governorate (60%), where the highest number of CCHF human cases were reported in 2022–23.

**Table 4.** CCHF serological PR (%) in cattles, buffaloes, sheep, and goats in comparison with human CCHF-confirmed cases in 2023 by Iraqi governorates^a^^,^^b^

		Cattle and buffaloes			Sheep and goats			All animals		
Governorate	CCHF human cases	Tested	Positive	IgG PR (%)	Tested	Positive	IgG PR (%)	Tested	Positive	IgG PR in animals (%)
Thi-Qar	142	197	121	61	121	70	58	318	191	60
Baghdad	90	243	33	14	65	9	14	308	42	14
Basra	86	97	36	37	48	7	15	145	43	30
Maysan	39	89	24	27	67	16	24	156	40	26
Wasit	39	155	90	58	172	88	51	327	178	54
Muthanna	35	40	27	68	137	18	13	177	45	25
Diwanya	24	194	70	36	119	44	37	313	114	36
Babil	23	276	101	37	54	20	37	330	121	37
Diyala	18	193	71	37	135	17	13	328	88	27
Najaf	17	75	21	28	132	43	33	207	64	31
Kirkuk	15	101	25	25	170	5	3	271	30	11
Erbil	14	57	18	32	25	8	32	82	26	32
Ninewa	13	126	50	40	575	3	1	701	53	8
SalahAl-Din	10	11	0	0	73	8	11	84	8	10
Al-Anbar	2	35	5	14	185	7	4	220	12	5
Kerbala	11	Nil^c^	Nil^c^	Nil^c^	Nil^c^	Nil^c^	Nil^c^	Nil^c^	Nil^c^	Nil^c^
Duhok	5	Nil^c^	Nil^c^	Nil^c^	Nil^c^	Nil^c^	Nil^c^	Nil^c^	Nil^c^	Nil^c^
Al-Sulaymaniyah	4	Nil^c^	Nil^c^	Nil^c^	Nil^c^	Nil^c^	Nil^c^	Nil^c^	Nil^c^	Nil^c^
Total	539	1889	692	37	2078	363	3.44	3967	1055	27

a Animal data source: Ministry of Agriculture Iraq/Central Veterinary Laboratory, *N* = 3967 domestic animals tested in 15 governorates for IgG seropositivity in 2023.

b Human data source: Ministry of Health Iraq/Communicable Disease Control Center, *N* = 539 CCHF-confirmed human cases reported from 18 governorates in 2023.

c Nil = data not collected and not available.

## Discussion

Since 2022, a drastic increase in CCHF cases has been reported in Iraq, with a 10- to 20-fold rise in cases compared to previous years. Data from 2023 compared to 2022 (Table 1 and Supplementary Fig. S3) show a continuous rise in confirmed cases and the spread of CCHFV across all 19 governorates, both rural and urban (Supplementary Fig. S4). A 27% decrease in CFR from 2022 may be attributed to enhanced response activities—for instance, enhanced tick control measures, improved surveillance, laboratory confirmation, case management, and risk communication—by the Iraqi government, WHO, and partners. Differences between rural and urban settings were noted, with women more likely to live in rural settings, exposure to ticks reported with animals at home, and tick bites more commonly reported from rural settings. In contrast, men were more likely to live in urban settings and have a history of slaughtering (Table 2 and Supplementary Fig. S4).

The most reported occupation was housewives (Table 2), with some implications as the collected data (shown in Table 2) describes. In Iraq, housewives often engage in various activities, particularly in rural areas, and they have frequent opportunities for being exposed to risks of contracting CCHFV. More studies are necessary to better document housewives’ routes of infection and propose appropriate measures to decrease their exposure to CCHFV.

The most frequently reported risk exposure history was contact with raw meat, followed by slaughtering, the presence of animals in the house, tick bites, and contact with suspected or confirmed CCHF cases (Table 2), with potential recall bias as data was collected through interviews with patients and/or their families. Additionally, the history of tick bites may be underestimated since tick bites are painless and often unrecognized, particularly in hyperendemic areas. Reporting tick bites might also be stigmatizing as a proxy for poverty. Furthermore, the literature generally states the main risks for CCHFV exposure as tick bites, slaughtering activities, and contact with other CCHF patients [2–9]. The true risks of “contact with raw meat” or “presence of animals in the house” require more research to determine whether they are more associated with direct virus exposure or behavioral risk factors for CCHFV infection.

Another finding includes the proportion of confirmed CCHF human cases: 42% female and 58% male, and the most affected age group among all cases: 25–44 years old (45%) (Table 3 and Supplementary Fig. S5). This suggests more frequent exposures to CCHFV occur among male and the most productive age group in the community.

Regarding the clinical presentations (Table 3 and Supplementary Fig. S6), the most common clinical manifestation was fever, followed by hemorrhagic symptoms. Bleeding from the gums or mouth and subcutaneous bleeding were more frequent in patients with a fatal outcome. More in-depth clinical studies should be conducted to analyze the differences in patients who died compared to those who survived, and more systematic clinical data collection is required to address whether the clinical presentations and their proportions are similar in different governorates. Further, to understand the drivers and factors affecting the reduced mortality in 2023 compared to 2022, the current CCHF treatment protocol and case management practice adopted in Iraq should be validated by an observational study with standardized clinical indicators, all medications and treatments used for patients, underlying diseases, and risk factors.

As detailed in Supplementary Fig. S7, the 2022–23 data demonstrate a distinct seasonality and trend in CCHF incidence in Iraq, with cases occurring predominantly between May and November, and peaking from May to July. The primary vector of CCHFV, ticks of the Hyalomma genus, thrive in the environmental conditions prevalent in southern Iraq, which are conducive to their habitation and reproduction [19]. Although the observed seasonal pattern of tick density appears to correlate with the incidence of CCHF cases in humans [19], further research is warranted. Specifically, studies focused on tick density across different governorates are necessary to elucidate the factors driving the heightened transmission of CCHFV to humans in, and the concentration of cases within, the southern governorates of Iraq.

Some reports [20, 21] claim an increased risk of CCHFV transmission during Eid-al-Adha as many people slaughter animals during this period. Eid-al-Adha for 2022 was celebrated on 9 July, and in 2023, it was on 28 June. However, this study could not observe a direct correlation in the increased number of CCHF cases during or after Eid-al-Adha, and the events were already in the CCHF high season. Iraq’s ongoing campaign for safe slaughtering, particularly before the Eid holidays, may also be contributing to this.

Concerning the study data on tick infestation in domestic animals (Supplementary Fig. S8), while tick infestation does not inherently indicate CCHFV infection in these animals, it underscores the need for more comprehensive studies to elucidate the implications of this high infestation rate. Such research is essential to better understand the potential zoonotic transmission dynamics and the associated risks of CCHFV infection in human populations [22, 23]. Moreover, a prospective study could be designed to establish definitive evidence of CCHFV transmission pathways from infected ticks to humans.

The comparative analysis between CCHFV serological PR in domestic animals and confirmed human cases by governorate (Table 4 and Supplementary Fig. S9) shows that the highest CCHFV serological PR among domestic animals was observed in the hyperendemic governorate of Thi-Qar, located in the south. More comprehensive seroprevalence studies by governorate, ideally for both animals and humans, should be conducted to understand the geographical differences in the risks of CCHFV infection to humans in Iraq, as CCHFV seropositive domestic animals were reported from all investigated governorates.

It is evident that there is a critical role of Hyalomma spp. ticks in human CCHFV infection. One study reports that rising temperatures influence the abundance of Hyalomma spp. ticks [23]; however, not many studies have been done on the factors affecting Hyalomma tick activities. To further investigate, we analyzed the trends in climate change in Iraq. The data were extracted from the World Bank site [18], which demonstrates that the average temperature has shifted by 2°C, from 21.63°C to 23.07°C between 1951 and 2020 in Iraq, with the same seasonal pattern by month (Supplementary Figs S10 and S11).

The recent climate data from Iraq suggests that rising temperatures in recent years have increasingly favored the habitat and reproductive cycles of Hyalomma spp. ticks, with a notable extension of their active period compared to previous years. This may also imply an earlier onset and later cessation of tick activity throughout the year, thereby extending the period of transmission risk for CCHFV to humans. However, a critical question remains: If tick population dynamics are the primary driver of the CCHF outbreak in Iraq, why has a comparable surge in CCHF cases not been observed in other countries experiencing similar climate shifts and temperature increases? Additionally, what factors contributed to the abrupt onset of the CCHF outbreak in Iraq in 2022, which continued to escalate in 2023?

A potential contributing factor could be the suspension of tick control activities in Iraq between 2020 and early 2022, due to COVID-19-related restrictions. The global pandemic, which disrupted routine animal dipping and tick control programs, may have facilitated the unchecked proliferation of CCHFV during this period. Tick control activities were reinstated after Iraq began observing a sudden surge in CCHF cases in early 2022. To collectively address these questions, it will be necessary to conduct comprehensive, multidisciplinary research that integrates the human, animal, and environmental health sectors.

## Conclusion

The increasing burden of CCHF in Iraq underscores the urgent need for proactive, cross-sectoral measures to mitigate its impact. The sharp rise in cases during 2022–23, the spread to urban areas, and the elevated mortality rate underscore the critical nature of the situation. Addressing these challenges necessitates a comprehensive One Health approach [24], incorporating enhanced research, robust surveillance systems, effective case management, public education, vector control strategies, and strengthened intersectoral collaboration. The correlation between human CCHF cases, tick population dynamics, animal seroprevalence, and climate change highlights the necessity of adopting this integrated approach and implementing stringent control measures to curb disease transmission.

Without the deployment of intensified interventions by both human and veterinary health sectors within the One Health framework [24–26], Iraq is likely to remain hyperendemic for CCHF. A coordinated, cross-sectoral strategic response is essential to prevent future outbreaks and protect public health in Iraq and the broader region. By implementing evidence-based strategies and fostering collaboration at the national, regional, and global levels, we can effectively combat the spread of CCHF and safeguard vulnerable populations from the significant threat posed by this disease.

## Supplementary Material

ckae147_Supplementary_Data

## Data Availability

The datasets generated and/or analyzed during the current study are available from the corresponding author upon reasonable request. Data sources include the Iraqi Ministry of Health, the Ministry of Agriculture, the World Health Organization, and the World Bank. Additional data are accessible in supplementary files and tables provided with this manuscript.
Key points Crimean-Congo hemorrhagic fever (CCHF) poses a significant global health threat, with an estimated 3 billion people at risk worldwide and fatality rates in humans up to 40%. CCHF outbreaks in Iraq have reached historic highs in 2022–23, with increased cases reported across urban and rural areas, indicating widespread transmission. Findings demonstrate the role of domestic animals and ticks in the transmission of CCHF, highlighting the importance of veterinary and public health collaboration for outbreak control. Global warming and climate change may be a driving factor in the increased incidence of CCHF, with rising temperatures favoring tick proliferation and extending their activity period. In response to the ongoing outbreak, Iraq has adopted a comprehensive One Health approach, integrating human, animal, and environmental health strategies to mount an effective CCHF prevention and control response in Iraq and neighboring countries.
