# Supplementary material for: Epidemiological analysis and potential factors affecting the 2022–23 Crimean-Congo hemorrhagic fever outbreak in Iraq
Source: Eur J Public Health. 2025 Jan 13;35(Suppl 1):i6–i13. doi: 10.1093/eurpub/ckae147 (PMC11725957; doi:10.1093/eurpub/ckae147)
Supplement: ckae147_Supplementary_Data [file ckae147_supplementary_data.pdf]

Supplementary Data

Epidemiological Analysis and Potential Factors Affecting the 2022-23 Crimean Congo Hemorrhagic Fever Outbreak in Iraq

Supplementary Figure S1: Global geographic distribution of Crimean-Congo Haemorrhagic Fever

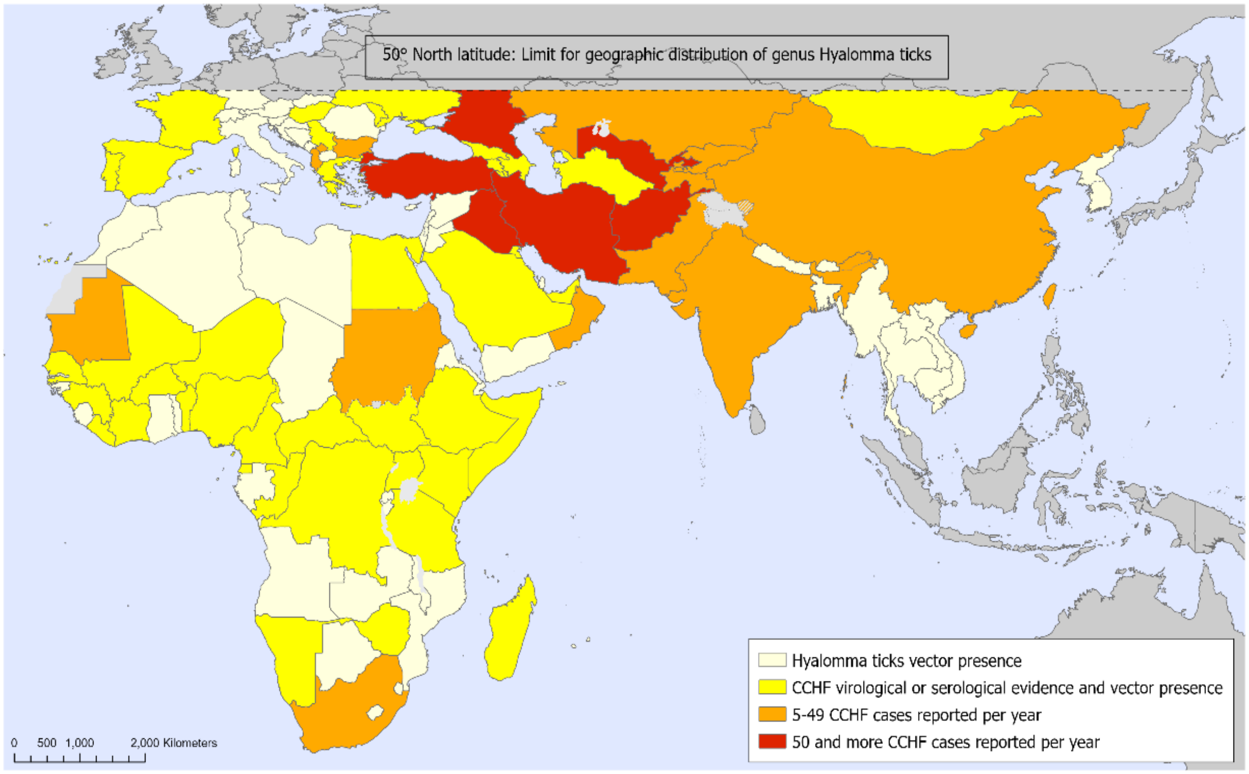

Data source: World Health Organization, 2022

Supplementary Figure S2: Number of CCHF confirmed cases in Iraq between 1986 and 2023

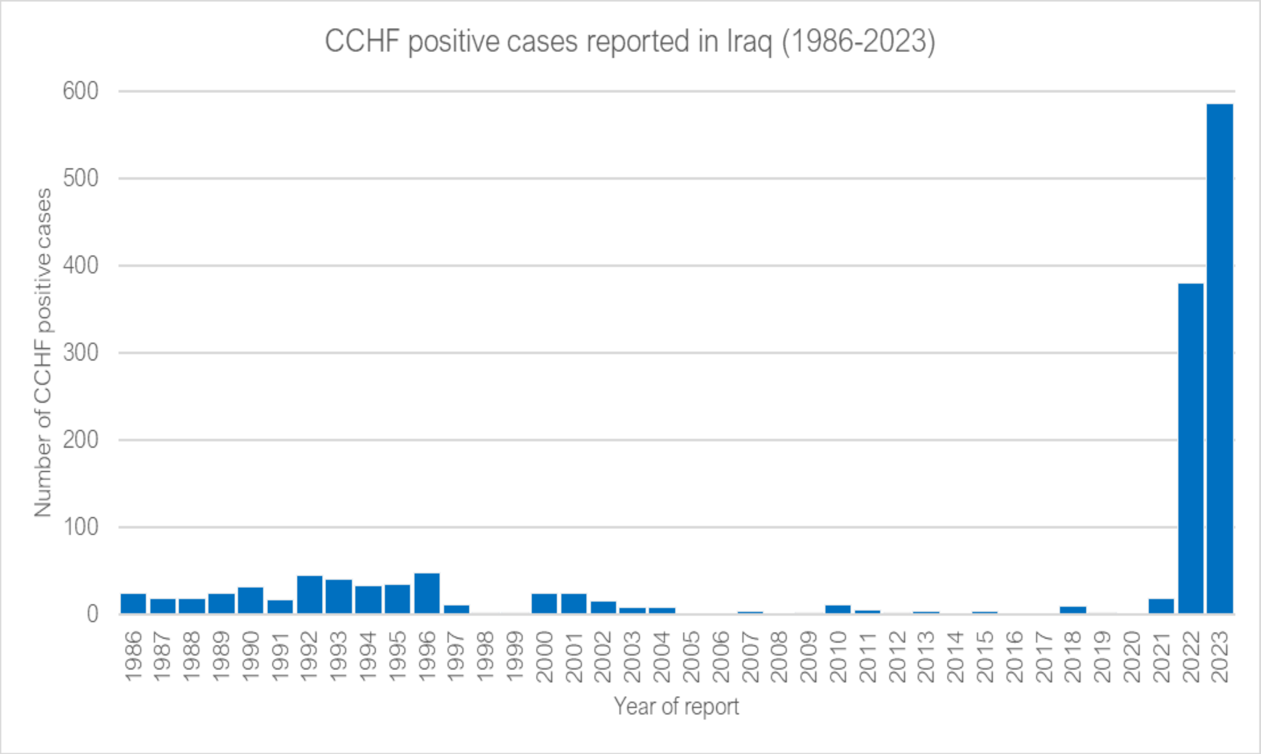

Source: Ministry of Health Iraq / Communicable Disease Control Center.

Supplementary Figure S3: Cumulative number of CCHF confirmed cases during 2022-2023 by Iraqi Governorates and outcome and case fatality rate (CFR)

N=967 confirmed cases reported from 01 January 2022 to 31 December 2023.

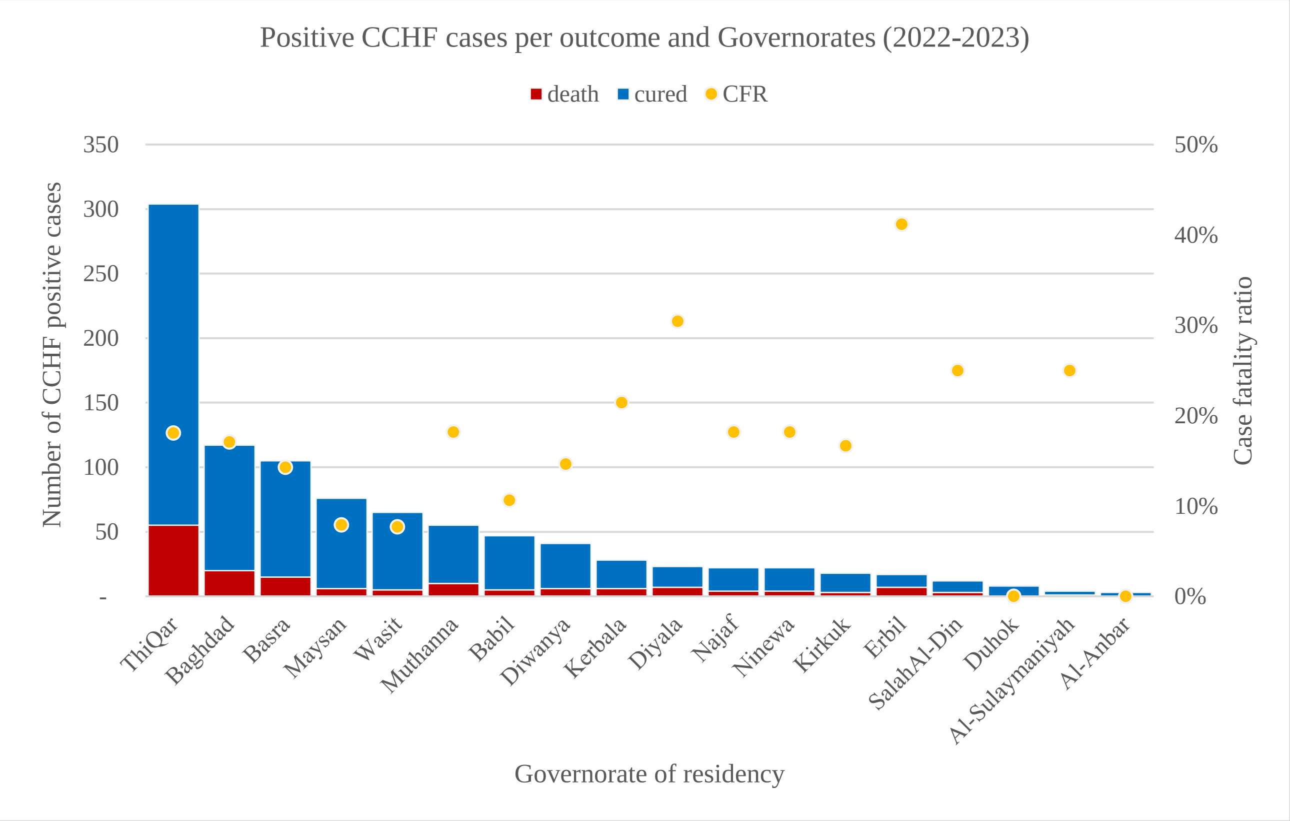

Source: Ministry of Health Iraq / Communicable Disease Control Center.

Supplementary Table/Figure S4: Proportion of CCHF cases per type of settings and by Iraqi Governorate

N=919 cases with type of settings reported. CCHF cases confirmed from 2022 to 2023.

		Distribution by settings							
**Governorate**	**Total cases**	**rural**	**Rural (%)**	**urban**	**Urban (%)**	**semi rural**	**Semi urban (**	**others**	**Others (%)**
ThiQar	293	162	55%	124	42%	7	2%		
Baghdad	111	30	27%	60	54%	15	14%	6	5%
Basra	100	51	51%	32	32%	13	13%	4	4%
Maysan	74	36	49%	34	46%	2	3%	2	3%
Wasit	61	34	56%	24	39%	3	5%		
Muthanna	54	40	74%	8	15%	6	11%		
Babil	44	30	68%	11	25%	3	7%		
Diwanya	35	25	71%	9	26%	1	3%		
Kerbala	26	9	35%	14	54%	2	8%	1	4%
Diyala	21	15	71%	4	19%	2	10%		
Najaf	22	9	41%	12	55%	1	5%		
Ninewa	19	11	58%	6	32%	2	11%		
Kirkuk	17	11	65%	6	35%				
Erbil	16	7	44%	4	25%	5	31%		
SalahAl-Din	12	5	42%	6	50%	1	8%		
Duhok	8	4	50%	1	13%	3	38%		
Al-Anbar	3	3	100%						
Al-Sulaymaniyah	3	3	100%						
**All**	**919**	**497**	**54%**	**362**	**39%**	**66**	**7%**	**13**	**0**

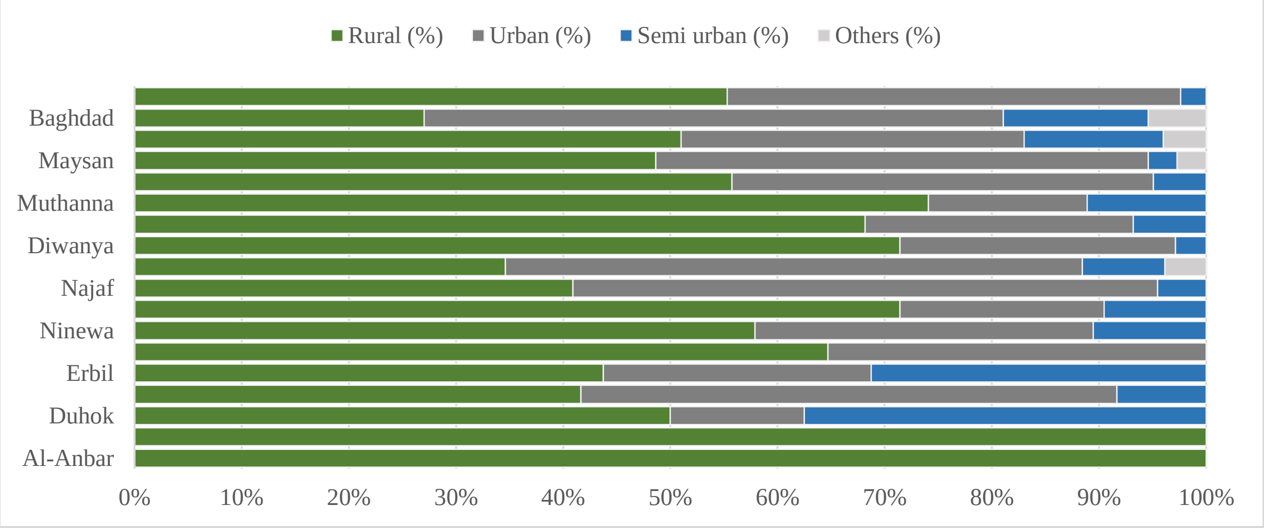

Source: Ministry of Health Iraq / Communicable Disease Control Center.

Supplementary Figure S5: Distribution of CCHF confirmed cases in Iraq by age and sex

N=919 CCHF cases reported from 2022 to 2023.

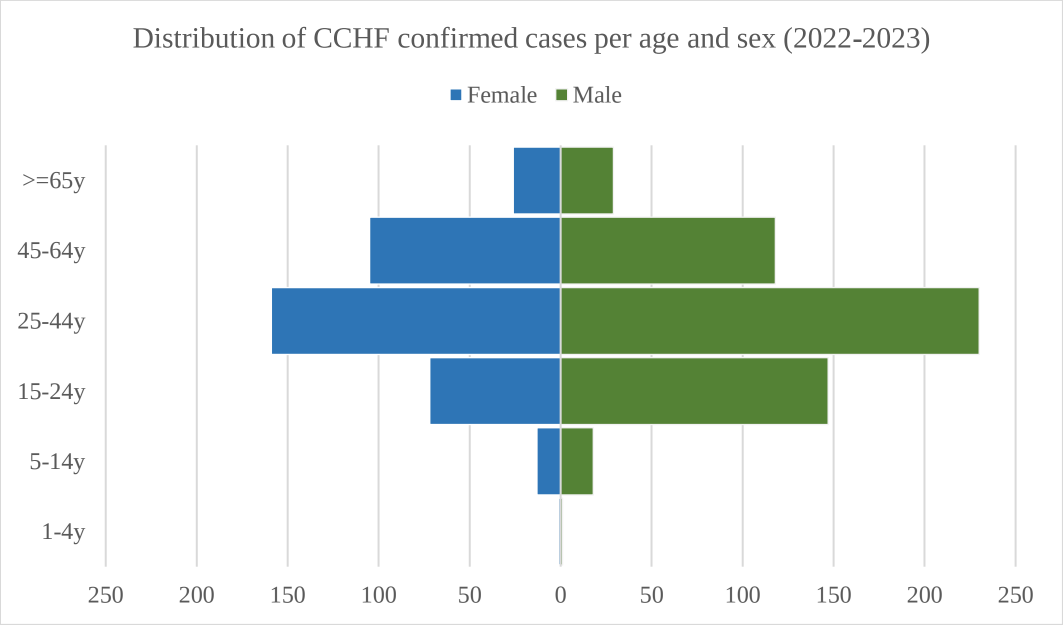

Source: Ministry of Health Iraq / Communicable Disease Control Center.

Supplementary Figure S6.1 and S6.2: Clinical presentations among CCHF recovered and deceased patients in Iraq

S6.1: Clinical presentations among CCHF recovered patients

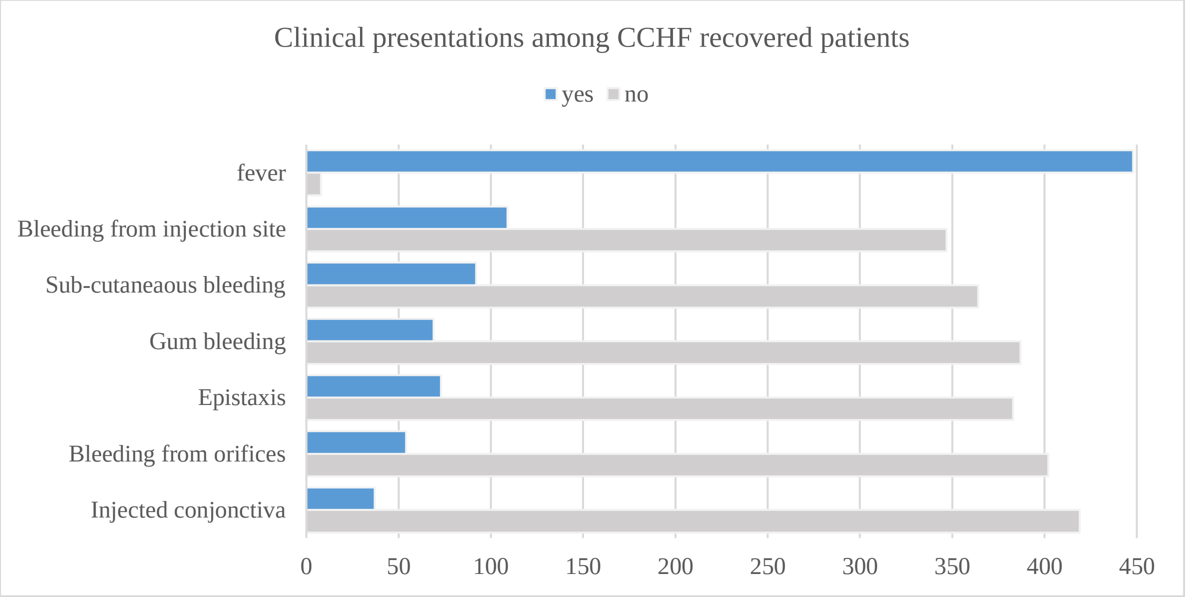

S6.2: Clinical presentations among CCHF deceased patients

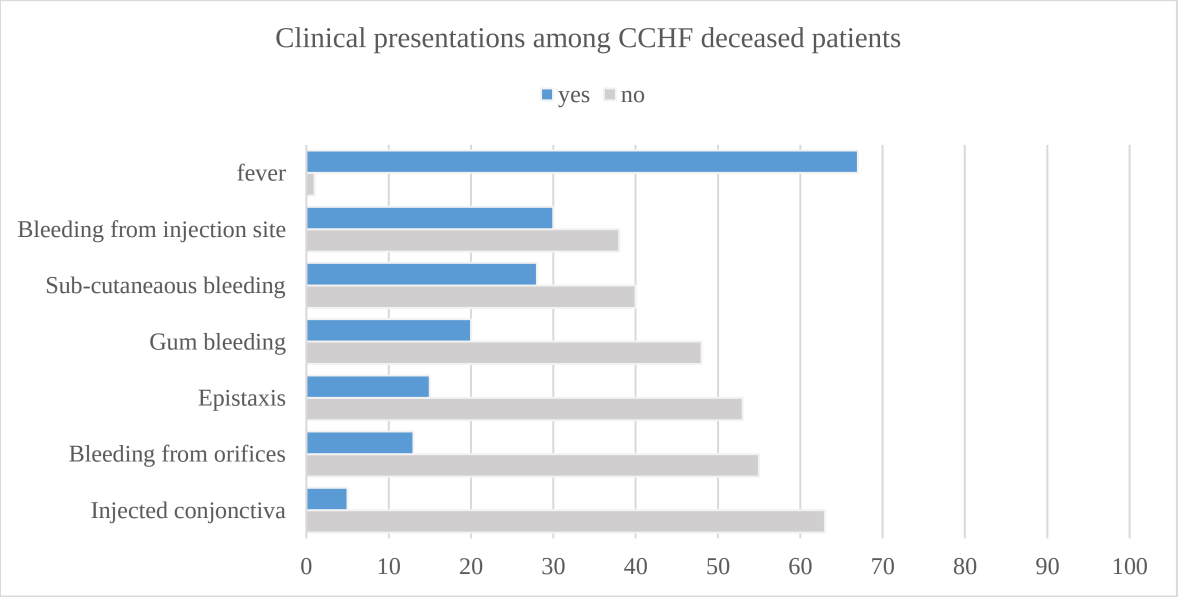

Supplementary Figure S7: Comparison of cumulative number of confirmed CCHF cases from 2007-2021 to year 2022 and 2023 in Iraq by month

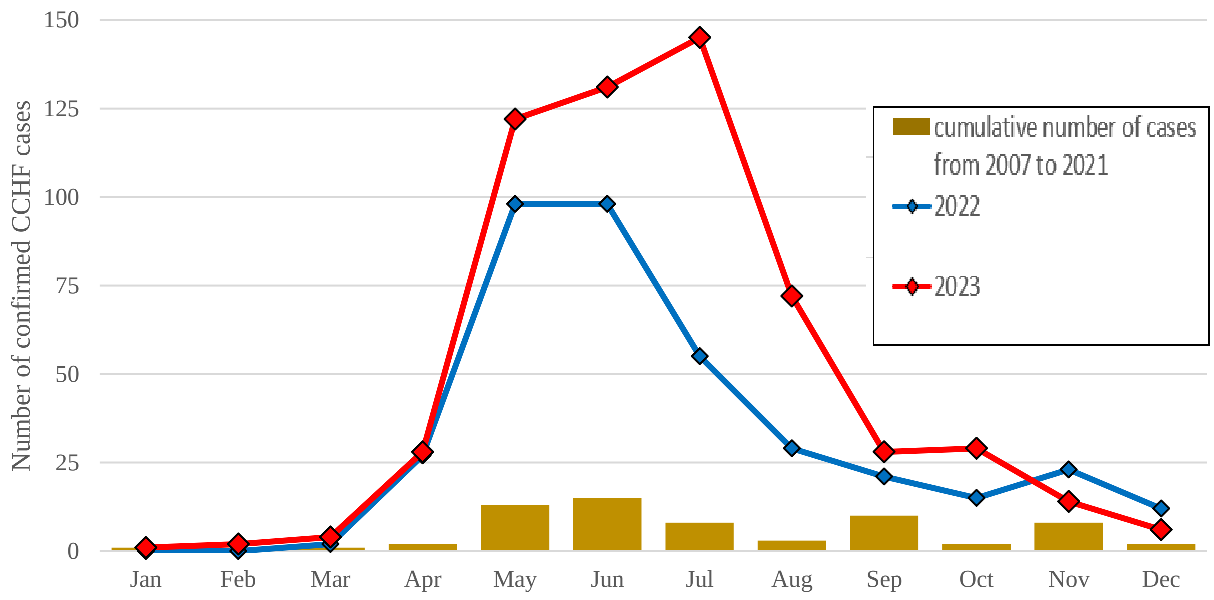

Source: Ministry of Health Iraq / Communicable Disease Control Center

Supplementary Figure S8: Percentage of Tick Infestation by Domestic Animal in Iraq

Figure Legend: Rates of tick infestation on various domestic animals in Iraq

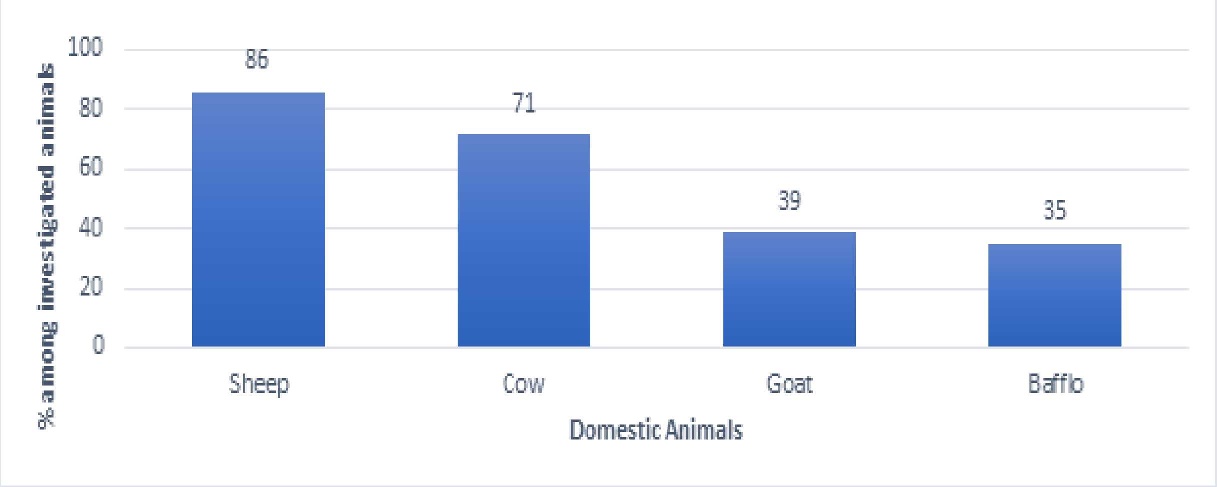

Source: Ministry of Health Iraq / Communicable Disease Control Center

Supplementary Figure S9: CCHF Seropositivity rate (%) in Domestic Animals in Comparison with CCHF Confirmed Human Cases by Iraqi Governorates

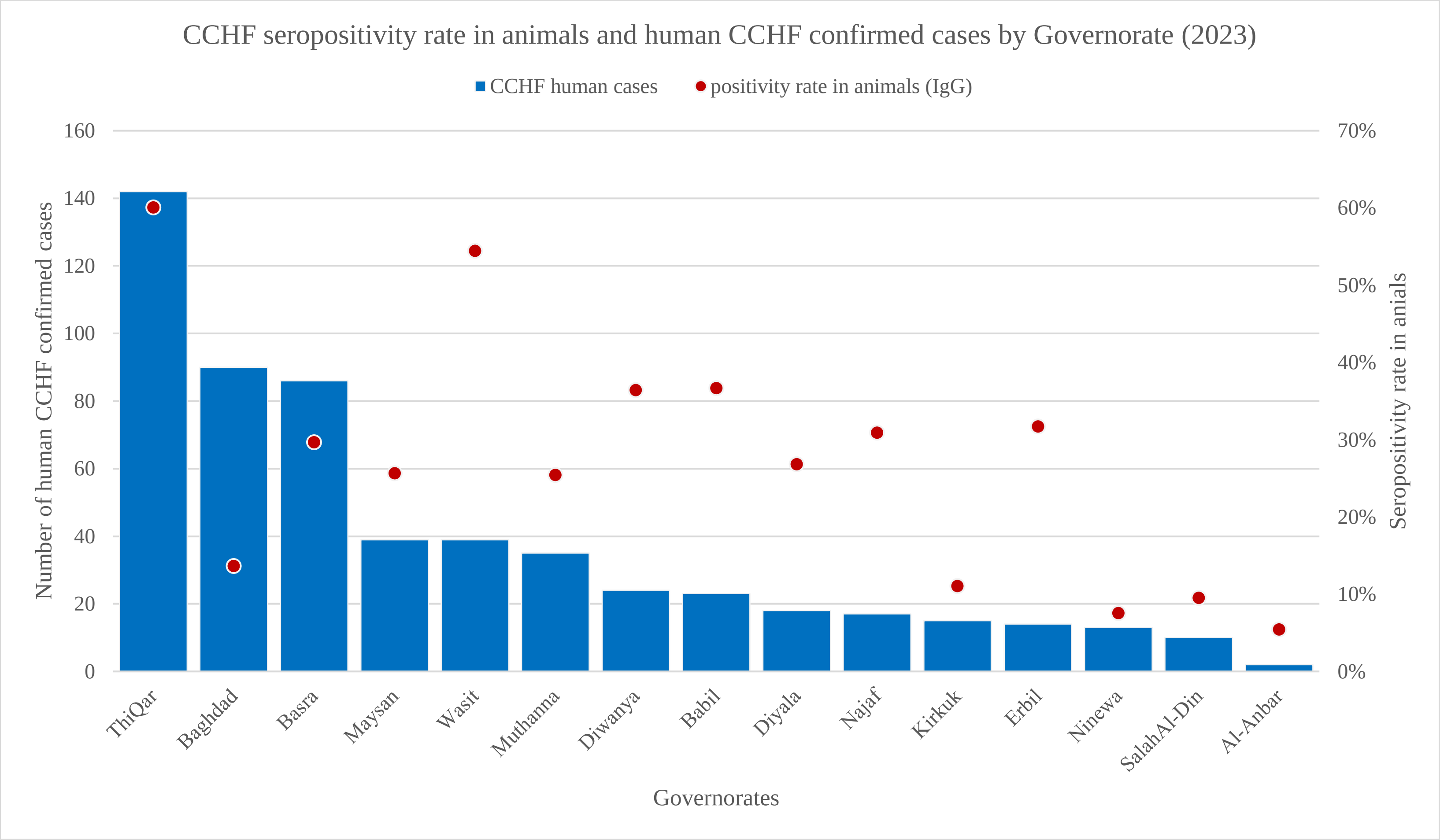

Source: Ministry of Health Iraq / Communicable Disease Control Center, Ministry of Agriculture Iraq / Central Veterinary Laboratory

Supplementary Figure S10: Trends in Average Mean Surface Air Temperature in Iraq (1951-2020)

Legend: This figure outlines the rising trend in average mean surface air temperature across three distinct periods: 1951-1980, 1971-2000, and 1991-2020 in Iraq.

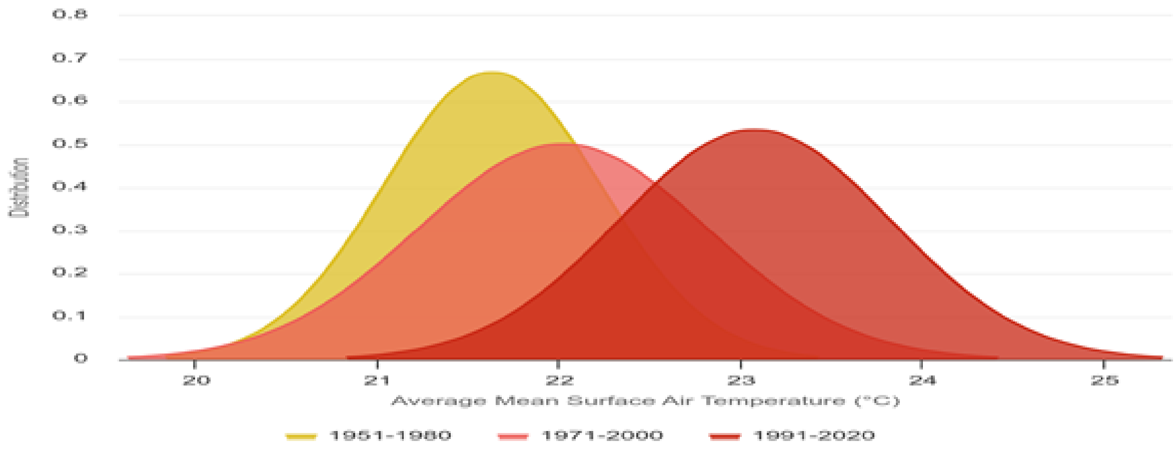

(Data source: Climate Change Knowledge Portal, The World Bank)

Supplementary Figure S11: Seasonal Variability and Trends of Average Mean Surface Air Temperature in Iraq (1951-2020)

Figure Legend: This figure depicts the month-by-month variability and trend of average mean surface air temperature over the years 1951-2020 in Iraq.

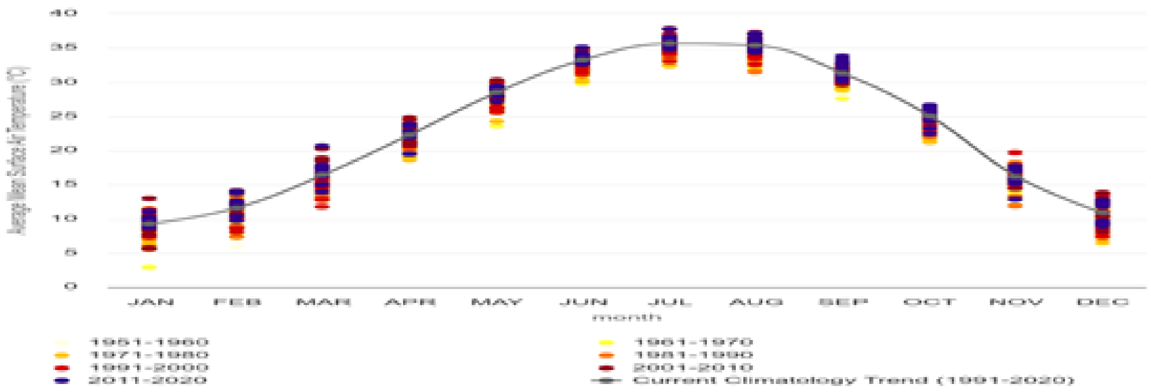

(Data source: Climate Change Knowledge Portal, The World Bank)
